# Nanofiltration for Arsenic Removal: Challenges, Recent Developments, and Perspectives

**DOI:** 10.3390/nano10071323

**Published:** 2020-07-06

**Authors:** TA Siddique, Naba K. Dutta, Namita Roy Choudhury

**Affiliations:** Chemical and Environmental Engineering, School of Engineering, RMIT University, Melbourne, Victoria 3000, Australia; s3642366@student.rmit.edu.au

**Keywords:** natural water, arsenic, arsenic toxicity, arsenic removal, nanofiltration process, nanofiltration membrane

## Abstract

Arsenic (As) removal is of major significance because inorganic arsenic is highly toxic to all life forms, is a confirmed carcinogen, and is of significant environmental concern. As contamination in drinking water alone threatens more than 150 million people all over the world. Therefore, several conventional methods such as oxidation, coagulation, adsorption, etc., have been implemented for As removal, but due to their cost-maintenance limitations; there is a drive for advanced, low cost nanofiltration membrane-based technology. Thus, in order to address the increasing demand of fresh and drinking water, this review focuses on advanced nanofiltration (NF) strategy for As removal to safeguard water security. The review concentrates on different types of NF membranes, membrane fabrication processes, and their mechanism and efficiency of performance for removing As from contaminated water. The article provides an overview of the current status of polymer-, polymer composite-, and polymer nanocomposite-based NF membranes, to assess the status of nanomaterial-facilitated NF membranes and to incite progress in this area. Finally, future perspectives and future trends are highlighted.

## 1. Introduction

In recent years, the source of pure water is continuously diminishing due to groundwater decline and depletion, climate change, poor resource management, and environmental pollution. In addition to this, the increasing rate of the world population, ~80 million per year, results in a growing demand for water by 64 billion cubic meters per annum [1,2]. The major pollutants of water are organic dyes, radioactive metals, heavy metals, and oxyanions of metals (CrO_4_^2−^, AsO_4_^3−^, SeO_3_^2−^, etc.). Among the pollutants, the toxicity of arsenic oxyanions is very serious and unique as they originate into the water body due to natural phenomena—either from natural soil sources or from anthropogenic sources. Moreover, they are carcinogenic. The consequence of consumption of arsenic-contaminated drinking water has evolved as one of the major health hazards in recent times. High concentration of arsenic (As) in drinking water may initially cause skin disease and eventually can turn to cancer and is of the utmost concern to public health [3]. The regulatory agencies around the globe such as World Health Organization (WHO) [4], US Environmental Protection Agency (USEPA) [5], Health Canada [6], and European Union (EU) [7] have set maximum limits on arsenic in drinking water to 0.01 mg/L to ensure the safe consumption of arsenic-contaminated drinking water to protect people from diseases. People of different parts of Bangladesh, West Bengal [8] and some other parts of India [9], Cambodia [10], Inner Mongolia of China [11,12], Iranian Kurdistan [13], Thailand [14], Eastern Croatia [15], Mexico [16], El Salvador, Peru, Nicaragua [17], northern Afghanistan, northern Mali and Zambia in Africa [18], and Vietnam [10] are living under the dire threat of arsenic toxicity [19,20]. At present, there are 628.5, 2281.2, and 2071.8 million people living in 21, 29, and 26 countries that are in the categories of the low, lower medium, and upper-medium groups respectively [21]. Among them, the poisoning effect of arsenic is most severe in the areas of Bengal Delta (Bangladesh, Nepal, and West Bengal), where concentrations of dissolved arsenic exceed over 200 μg/L. More than 3.57 billion people living in Bangladesh, Vietnam, Pakistan, India, Afghanistan, Nepal, Mali, Nigeria, etc. are at risk, and due to the arsenic-contaminated drinking water, at least 100 million people are already affected [20]. Consequently, effective purification methods for arsenic removal from water is of critical importance and of profound societal value. However, due to the low socio-economic status of the major affected countries, they cannot afford expensive and large-scale treatments to remove arsenic from drinking water to the acceptable levels (10 ppb, as recommended by WHO and US EPA). Therefore, development of facile and cost-effective methods, which are easy to handle and conveniently applied in large scale at household and community levels for the remediation of arsenic from contaminated groundwater is of the highest priority.

Considering the societal value and critical importance, both traditional and different emerging technologies based on chemical, biochemical/biological/biosorption, and physico-chemical treatment processes for arsenic removal from contaminated drinking water have been developed and applied [22]. Many sustainable and naturally abundant materials including waste rice husk [23]; iron-based adsorbents such as iron oxy-hydroxides including akaganeite (β-FeOOH), goethite (α-FeOOH), lepidocrocite (γ-FeOOH), ferrihydrites (Fe_10_O_14_(OH)_2_), iron-based layered double hydroxides (LDHs), iron oxy-hydroxides doped activated carbon, and iron oxy-hydroxides doped graphene oxide [24,25]; and activated carbon [26] have been examined as efficient adsorbents for As removal. Recently, some novel materials including cellulose-based fibers [27,28], metal organic framework (MOF) [29], and hydrogel [30] have also been explored. Several of these As removal materials and techniques have also been implemented practically in the affected rural areas. The main concern of all the effective processes is mainly related to cost, both initial and operational, as the arsenic problem is mostly in developing or underdeveloped countries and areas. In a report by the World Bank, it has been stated that especially low (US$ < 1025), lower medium (US$ 1026–4035), and upper-medium (US$ 4036–12,475) income countries are facing challenges to reduce arsenic below the guideline values due to their limited economic capacities [31]. Therefore, there is a drive for continuous improvement in the existing removal method as well as introducing new technologies. 

The developed methods for ‘As’ removal include chemical precipitation, adsorption, coagulation, electrocoagulation, ion exchange, oxidation, and membrane filtration. The membrane filtration is a boosting technology in the field of separation technology. In recent years, potable water purification using membrane technology has become attractive worldwide due to its simplicity and versatility, increased stringency of regulations, decreasing costs, and increasing commercial availability of a variety of membranes. It does not require any additives or chemicals, and only minimal amounts of energy. Membrane technology is a clean technology, where the separation process is carried out solely on the basis of molecular size and use of additives is not necessary and has the potential to boost the product quality and bring down the overall production costs. Currently, it is considered superior to other separation methods because of its easy operation and no sludge formation. However, there are liquid wastes containing dissolved solids (concentrates) in the water rejected by membrane systems. The disposal of waste streams generated by removing arsenic from drinking water is of concern, because As can be highly mobile and has the potential to leach back into the ground and surface waters. The residual disposal options are highly site-specific. These wastes can be discharged to a receptor body if they comply with the regulations; on the contrary, they must be submitted to treatments such as chemical precipitation and coagulation-sedimentation-filtration with the generation of solid wastes (muds or sludges). In the case of semiliquid wastes, they must be submitted to thickening and dewatering processes [32]. Though, different effective, reliable, and sustainable methods of As waste disposal have been developed and proposed, recent emphasis has been given on stabilization/solidification (S/S) technologies, which are currently used to treat industrial wastes containing As [33]. 

The four most popular pressure driven membrane filtration processes for liquid separation are respectively: (i) reverse osmosis, (ii) nanofiltration, (iii) ultrafiltration, and (iv) microfiltration—in ascending order of size of the particle that can be separated. The microfiltration is a low pressure-driven filtration process for arsenic removal; however, these membranes are not highly efficient for arsenic removal as they are based on the pore flow model and allow multivalent ion to pass through the membrane pores. On the other hand, reverse osmosis membrane can efficiently remove arsenic; however, it is energy intensive and its operational cost is higher. Nanofiltration has attracted considerable attention from researchers due to its high efficiency with lower operational cost [34]. The separation process in nanofiltration (NF) membrane depends both on size sieving and Donnan exclusion, and efficiently rejects multivalent dissolved ions of water. These properties make it a highly suitable separation technology for arsenic removal [35].

In this review, first we briefly discuss the origin and mechanism of contamination of water with arsenic of different oxidation states. Next, we briefly discuss the conventional technologies currently being used for arsenic removal, which is followed by specific focus on the various types of recently developed and emerging nanofiltration membrane technology for arsenic removal. Finally, the synthesis methods for nanofiltration membrane and the effect of process parameters on their quality and performance will be examined. The advantages and disadvantages of the NF-membrane technology relative to other methods and future perspectives will also be presented.

## 2. Arsenic in Natural Water: Sources, Speciation, and Mobility

**Figure 1:**
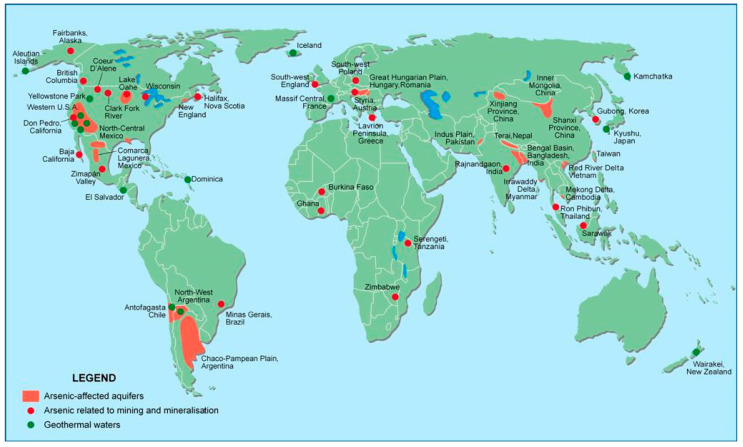
Arsenic affected aquifers as well as water and environmental problems related to mining and geothermal sources (areas in blue are lakes) (adapted with permission from [36]).

Arsenic is a metalloid that possesses characteristics of both a metal and a non-metal and is widely distributed in the soil, water, air, and rocks. Arsenic is an inevitable part of the natural water resources of the affected areas as the earth’s crust contains a large amount of arsenic. Several natural and anthropogenic sources have been identified to be responsible for As contamination in groundwater. As is at the position of 20 among the most abundant elements on earth. As occurs as a major constituent in more than 200 minerals and arsenopyrite (FeAsS) is the most abundant As containing mineral. Realgar (As_4_S_4_) and orpiment (As_2_S_3_) represent two common reduced forms of As; while As is also present in oxidized forms such as arsenolite (As_2_O_3_) [36]. As is released into water resources because of natural and anthropogenic activities such as the dissolution of minerals and complexion of natural organic matter [24]. The main anthropogenic sources for contamination of groundwater with As are mining, burning of fossil fuels, use of arsenical fungicides, herbicides, and insecticides in agriculture and wood preservatives [37]. Discharge of arsenic-contaminated water from industry and disposal of arsenic chemicals may also cause arsenic contamination in drinking water. Burning of coal has significant effect on the contamination of As in the environment (volatilization of As_4_O_6_ due to burning of coal). However, it is important to note that the As in water from anthropogenic sources is much less compared to the natural sources. Arsenic-affected aquifers as well as water and environmental problems related to mining and geothermal sources are distributed all over the world, as illustrated in Figure 1.

The status of As contamination in natural ground water in various countries is given in Table 1. High concentration of As in groundwater in a range from 1 to 73.6 mg L^−1^ is found in many countries including Bangladesh, India, China, America, Argentina, Chile, and Mexico [38]. A high concentration of As is also observed in surface water, as presented in Table 2.

**Figure 2:**
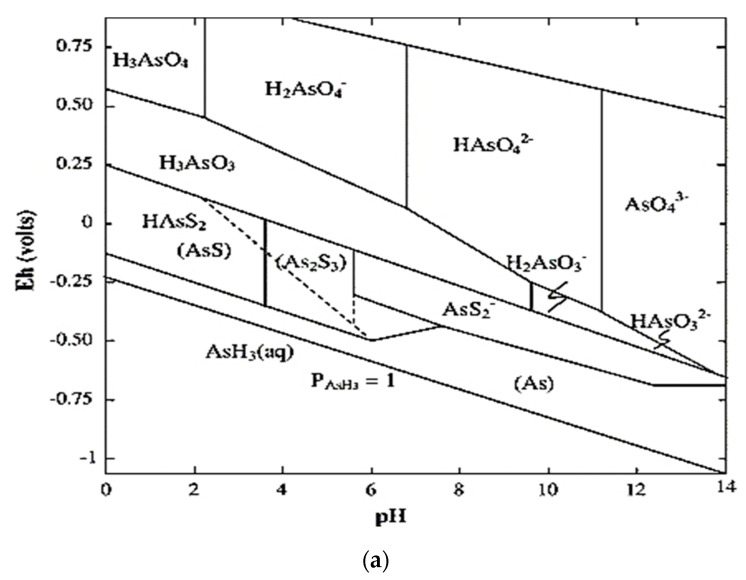

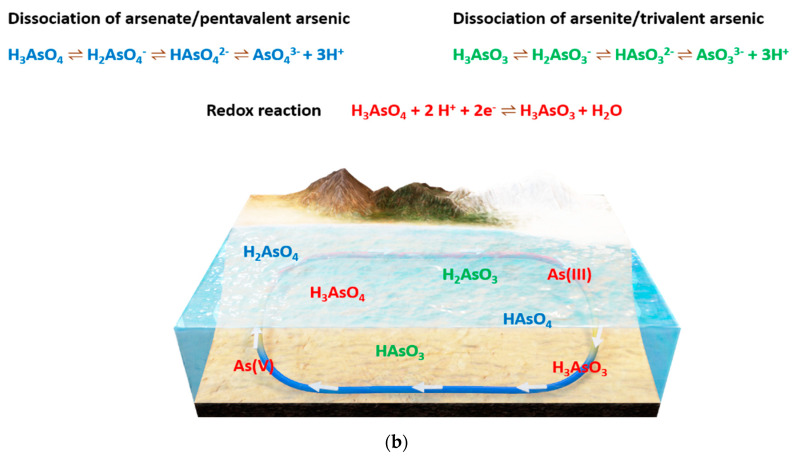
(**a**) The Eh–pH diagram for arsenic at 25 °C and 101.3 kPa (adapted with permission from [58]). (**b**) Arsenic in different oxidation state in natural water.

As presents in water in different oxidation states, most commonly as arsenic As^III^ (arsenite) and As^V^ (arsenate) form. The predominant As^III^ species are uncharged H_3_AsO_3_, while the primary arsenate species are monovalent H_2_AsO_4_^−^ and divalent HAsO_4_^−2^. The Eh–pH diagram, which illustrates the fields of stability of mineral or chemical species in terms of the activity of hydrogen ions (pH) and the activity of electrons (Eh), for arsenic is shown in Figure 2a. The transformation of states illustrated on Eh–pH diagrams involve either proton transfer (e.g., hydrolysis) or electron transfer (oxidation or reduction) or both. The arsenite and arsenate forms dissociate in water into oxy anions of arsenic, which are shown in Figure 2b. Under oxidizing conditions, inorganic arsenic is predominantly present as As^V^. H_2_AsO_4_^−^ is the most stable species between pH 2 and 7, while HasO_4_^−2^ is the most stable species above pH 7. H_3_AsO_3_ is the predominant species within the normal soil pH range (pH 4 to 9). As^V^ can be reduced to As^III^ under moderately reducing conditions (Eh~100 mV), which can be induced by flooding [57].

## 3. Toxicity and Health Risk of Chronic Arsenic Exposure

Among the different types of naturally occurring arsenic, inorganic arsenic is most prevalent. In nature ‘As’ is mostly available in the form of sulphides, oxides or salts of calcium, copper, sodium, iron, etc., which are toxic and is a major environmental and health concern in different parts of the world [59]. The toxicity of arsenic varies across its different forms. Inorganic arsenic compounds are more toxic than organoarsenicals, and trivalent arsenite is more toxic than pentavalent arsenate. Particularly, the oxyanion forms, arsenite As^III^ and arsenate As^V^ compounds are deadly to the living creatures as well as to the environment. The toxicity of arsenic is pH-dependent (Figure 2a). As^III^ is present as H_3_AsO_3,_ which is a neutrally charged oxyanion when the pH range is below 9.2 (pKa = 9.2). On the other hand, the species of As^V^ remains as negative oxyanions in physiological conditions (pH~7.4). The mobility and toxicity of As^III^ is higher than As^V^ as neutral ion decrease its probability of adsorption in the mineral surface [60]. In general, inorganic As being more toxic than organic As.

**Figure 3:**
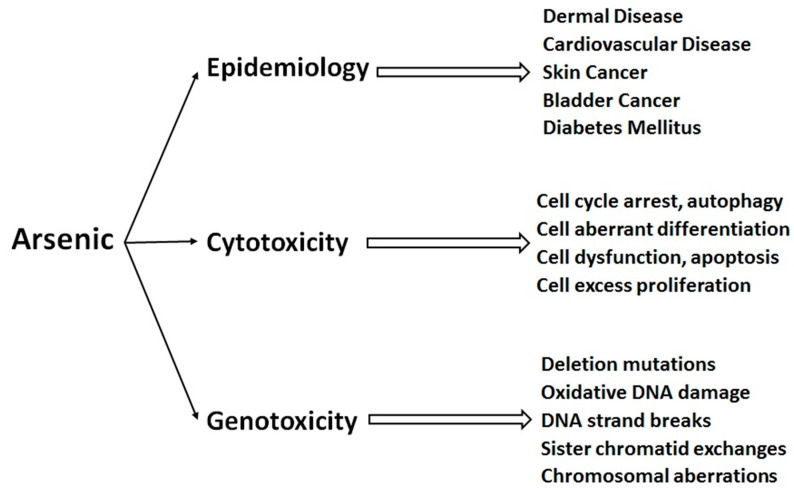
Arsenic toxicity in humans (adapted with permission from [67]).

Human exposure to arsenic occurs through the oral, respirator, or dermal routes. As consumption in the human body mostly occurs through direct consumption of the contaminated water; soil, contaminated agricultural and fish products may also be the source of As exposure. Exposure to organoarsenicals normally occurs through the consumption of contaminated marine animals and plants. Children’s health may be affected severely with the deliberate consumption of arsenic [61]. The mechanism of arsenic-induced toxicity is complex, because it is altered by its oxidation state and solubility, in addition to numerous other internal and external factors including exposure amount, length and frequency, the biological species, age, sex, individual sensitivity, genetics, and nutritional factors. Therefore, the precise mechanisms, that regulate acute or chronic exposure to arsenic are not yet completely recognized. It is considered as a protoplasmic poison because it affects the sulfhydryl group of cells and eventually causes malfunctioning of mitosis, cell enzymes, cell respiration, and accumulation of pyruvic acid in the blood. There is also interference in the normal enzymatic activity of the body and cell metabolism due to the consumption of arsenic. As^III^ can be transported into cells through aquaglyceroporins (proteins that are permeable to glycerol as well as water), whereas As^V^ entrance occurs via phosphate transporters. It has been proposed that following entry into the human cells, As^V^ rapidly reduced to As^III^. Thereafter, As^III^ undergoes multistep pathway of arsenic metabolism in cells through arsenite methyltransferase (AS3MT) using S-adenosylmethionine (SAM) as the methyl donor, resulting in the formation of methylated As compounds; including monomethylarsonous acid (MMA^III^), dimethylarsinous acid DMA^III^, monomethylarsonic acid (MMA^V)^, and dimethylarsinic acid DMA^V^ [62]. Biomethylation of inorganic arsenic, particularly the production of trivalent methylated metabolites, is a process that activates arsenic as a toxin and a carcinogen. Some metabolites are not excreted and remain inside the cell as an intermediate product, which are found to be highly toxic compared to other arsenicals, potentially accountable for arsenic-induced carcinogenesis. [59,63]. The toxicity of various arsenic species increases in the order of As^V^ < MMA^V^ < DMA^V^ < As^III^ <MMA^III^ ≈ DMA^III^. Schematic representation of the toxicities of arsenic in human health is shown in Figure 3. Several review articles have documented the toxicity of As in human health (Figure 3). It is a well-established fact that As induces epidemiological toxicity and chronic exposure to As can lead to arsenicosis, including skin lesions, blackfoot disease, and peripheral vascular disease. As is a potent carcinogen, leading to skin, bladder, liver, and lung cancers [64], and it also causes cytotoxicity and genotoxicity [65]. Arsenic exposure from drinking water has also potential to affect the reproductive system and pregnancy complications [66].

## 4. Nanofiltration for Arsenic Removal

### 4.1. Arsenic Removal Technologies from Drinking Water

Arsenic cannot be degraded but can only be separated from water or converted from aqueous phase to another phase, appropriately to solid. Removal of arsenic from drinking water is a matter of severe concern due to its vigorous harmful effect in the human body. Several researchers around the world are focusing on the issue by developing simple and low-cost technology to remove As from aqueous solutions, mainly drinking water (ground water and surface water).

**Figure 4:**
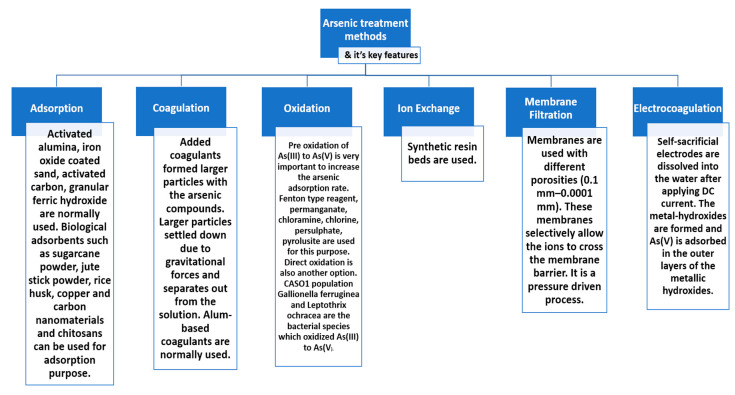
Established arsenic treatment methods with its key features.

Over the past decades, several methods have been investigated to remove As from water, which are mainly focused on the separation techniques including oxidation [68], coagulation [69], adsorption [70], ion exchange [71], electrocoagulation [72], and membrane filtration [73]. Recently, Nicomel et al. [74] presented an excellent review on the classical methods to remove As from water and will not be the focus of this article. However, the key features of some of the important As treatment methods are presented in Figure 4 and the advantages and the disadvantages of the methods are summarized in Table 3.

**Figure 5:**
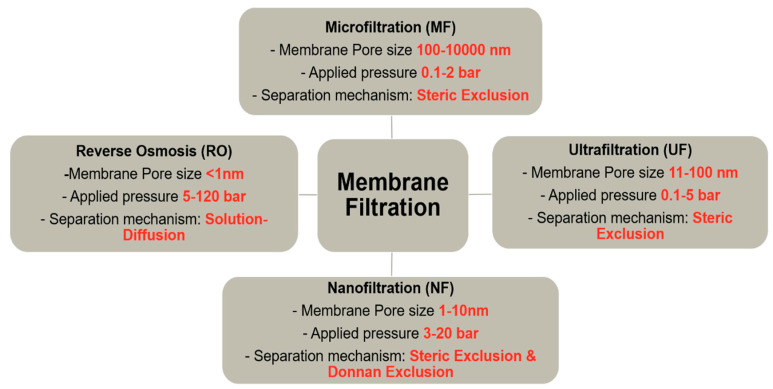
Distinguishing features of Microfiltration (MF),Ultrafiltration (UF), Nanofiltration (NF), and Reverse Osmosis (RO) process.

Membrane filtration technology has been established as the most efficient and desirable removal technique for arsenic from water [127] due to its advantages, including easy operational technique, high-removal efficiency, and no sludge production [128]. However, some unavoidable matters such as membrane fouling, flux reduction, and the discharge of the concentrate also need to be dealt with [24]. The disposal of waste streams of nanofiltration (NF) is not as problematic as that of a low-pressure membrane (micro-, ultra-filtration membrane) because the waste stream is less and not concentrated with dissolved ions. The filtration through a membrane involves the transport of the desired element under the influence of concentration and/or pressure gradient, and electrical potential. Figure 5 illustrates the different membrane filtration processes along with separation mechanism and operation conditions. The well-established membrane-based filtration processes are: microfiltration (MF), ultrafiltration (UF), nanofiltration (NF), reverse osmosis (RO), and electrodialysis. MF, UF, NF, and RO are pressure driven process and the pore diameter has a significant influence on the separation mechanisms and energy requirement. Another type of membrane filtration technology, which is known as electrodialysis can remove both arsenic and other contaminants, but the deposition of insoluble coagulants on the cathode is a major disadvantage [92]. Among all the filtration processes, RO and NF have been identified as the most efficient and reliable technologies, and they are useful for household applications, which is one of the most important criterions for drinking water purposes. Additionally, most of the dissolved salts can be removed efficiently using RO and NF [89,106,116,117,118,119,120,121,122,123,124,125,126]. RO membranes generally have a higher desalting ability but a lower water permeability than NF membranes. The NF process is considered prominent due to its lower initial cost, lower operational pressure, less consumption of energy, less membrane areas, and high removal efficiency of arsenic [129]. Therefore, NF is receiving significant recent attention, adaptation, and is growing in popularity. The major focuses have been on the novel material of choice, method of synthesis of the membrane, and their optimization. The rest of the review will be focused on the recent trends in the field of As removal by the nanofiltration process.

### 4.2. Nanofiltration Process

**Figure 6:**
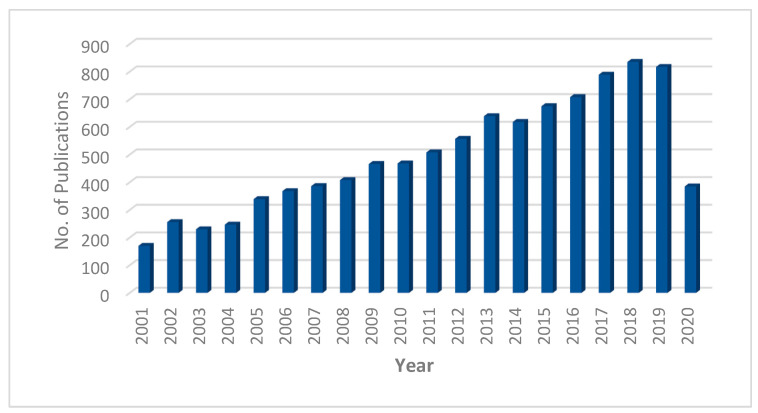
The number of publications each year since 2001 based on the keyword “nanofiltration” in Scopus database (data collected on 27.04.2020).

**Figure 7:**
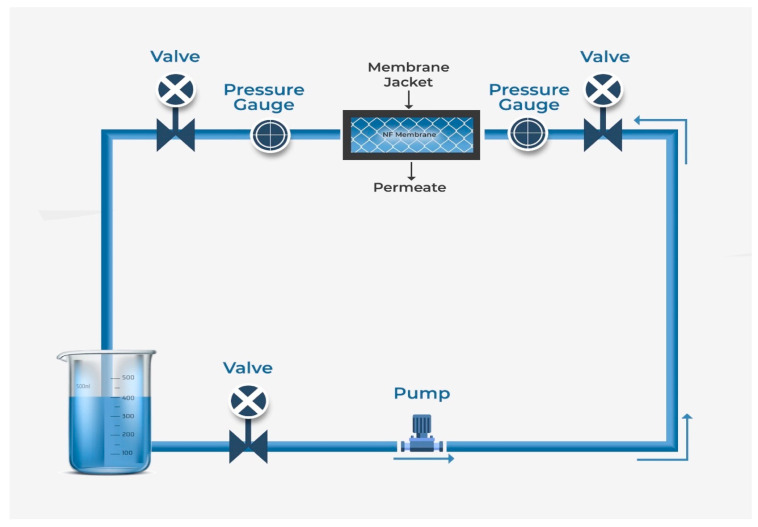
A schematic diagram of a typical nanofiltration process.

Nanofiltration (NF) is the most recently developed pressure-driven membrane process for liquid-phase separations. NF is a highly efficient, low-energy pressure-driven separation membrane technology for effective removal of low molecular weight solutes, such as salts, glucose, lactose, micro-pollutants, and natural organic matter in contaminated water [130,131]. NF membrane was first introduced in the late 1980s, and since then has gone through significant progressive transformation with time, which is shown in Figure 6 through a statistical trend in the number of publications per year for the last 20 years. In many applications, NF has replaced RO due to its lower energy consumption and higher flux rates. As shown in Figure 5, the characteristics of NF is between that of ultrafiltration (UF-separation occur due to size exclusion and, in some cases, charge effects) and reverse osmosis (RO-transport is governed by a solution diffusion mechanism). Nanofiltration membranes are categorized as having a through-pores in the size range of 10 to 1 nm and are near the range to remove rather small ions (the atomic radius of a sodium ion and a chlorine ion is about 0.97 and 1.8 nm respectively). The typical properties of NF membranes are: pore size ~2 nm, water permeability 5–50 Lm^−2^h^−1^bar^−1^, operating pressure 2–10 bar, molecular weight cut off >100 Da (generally 300–500 Da) [132]. A schematic diagram of a typical NF process is shown in Figure 7. NF shares many characteristics with RO. However, filtration through NF is an extremely complex process and is dependent on the micro hydrodynamic and interfacial events occurring at the membrane surface and within the membrane nanopores. In NF, the ion exclusion does not only depend on the porosity but also on the charge of the membrane.

NF combines hybrid function of both ultrafiltration and reverse osmosis membrane, which is also called loose reverse osmosis membrane. The key factors that control membrane performance in arsenic removal using NF includes porosity, charge, concentration polarization at the membrane face; and fouling of the membrane, concentration, and oxidation state of arsenic and membrane module. Therefore, the removal mechanism cannot be predicted simply based on the pore size [133].

### 4.3. Rejection Mechanism of Arsenic through NF Membranes

**Figure 8:**
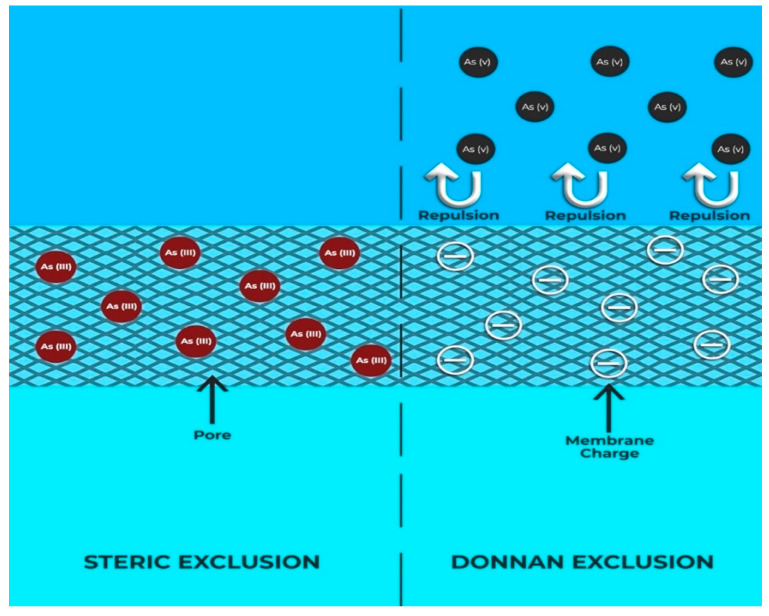
Separation mechanism of As through NF membrane as per pore size and Donnan exclusion.

NF membranes are usually applied to separate multivalent ions from monovalent ions and have great potential in removing arsenate from contaminated water. NF membranes that are commercially available have different levels of water permeability and As rejections. It has been found that water permeability of NF is not simply determined by membrane pore size (steric exclusion) and follow a more complex relationship and could be affected by surface hydrophobicity, roughness, and active layer thickness and charge. Most of the commercial NF membranes possess a fixed charge, which is developed by dissociation of surface groups such as sulphonated or carboxyl acids and allow ions to be separated by a combination of the size, electrical effect, and ion interaction mechanisms (Figure 8). It has also been identified that a more negative surface charge is favorable for arsenate rejection at circumneutral pH and the charge interaction plays a dominant role in separation of molecules using this membrane [134]. Rejection from NF membranes are attributed to a combination of steric, Donnan, dielectric, and transport effects [135]. The classical Donnan effect describes the equilibria and membrane potential interactions between a charged species and the interface of the charged membrane [136]. As depicted in Figure 8, the movement of neutral solutes through confined space of nanopores is via the steric mechanism, where the transport is greatly affected by the local environment; and the overall hindered transport is linked to both convective and diffusive element [137]. Whereas, the surface charge of the membrane rejects the arsenate ions because of Donnan effect [34]. According to Donnan effect, when a charged membrane is brought in contact with an electrolyte solution, the concentration partition of the counter ions and the co-ions occurs at the membrane solution interfaces on both the feed and the permeate sides. Donnan exclusion principle outlines that in an electrolyte solution the co-ions are excluded from the membrane matrix and counterions with a higher valence are preferentially adsorbed. For counterions with equal valence, the ions with the highest polarizability or with the smallest hydration shell are preferred to be adsorbed. The charge species that are allowed to cross the membrane (counterions) will equilibrate across the membrane until the ‘Donnan equilibrium’ conditions. 

The presence of charge on membrane/membrane pore surfaces originates from the dissociation of the ionizable groups at the membrane surface and from within the membrane pore structure [138]. Depending on the specific nature of the materials used during the fabrication of the NF, the surface groups may be acidic/basic in nature or may be a combination of both. The dissociation of these surface groups is strongly influenced by the pH of the contacting solution. The phenomena of dielectric exclusion in NF are much less understood and there are conflicting theories, as discussed in detail elsewhere [139]. NF membranes are usually asymmetric and negatively charged at neutral and alkaline media but lose their charge in acidic pH. This type of membrane can remove both dissolved arsenate and arsenite due to size exclusion according to the report by the EPA in 1999 [125]. Many studies have demonstrated that arsenic can be excluded even at a very high concentration of oxyanion of arsenic using a membrane without increasing the transmembrane pressure. The rejection is prominent for negatively charge arsenate relative to neutral arsenite if the membrane is negatively charged. Donnan exclusion is very effective when the concentration of co-ions in the aqueous solution is low and that of fixed charges on the membrane is high. During arsenic exclusion, the sieving mechanism and Donnan exclusion both can be active depending on the pH and membrane materials. As an example, the polyamide-based NF membrane possesses negative zeta potential at higher pH [140]. The Donnan effect is dominant when the membrane becomes negatively charged at high pH as the separation of ionic species depends on charge repulsion. As arsenic remains in water in different oxidation states, the removal mechanism may not be always Donnan effect dominant. 

The nano-scale phenomena involved in ion separation by NF are complex and many models have been advanced for macroscopic description of the ion transport and partitioning. The initial descriptions of ion transport in NF were based upon phenomenological equations defined through irreversible thermodynamics [141]. Such an approach has been employed successfully to predict the separation performance in NF; however, phenomenological models do not characterize the structural and electrical properties of the membranes. Another approach for description and modelling of realistic NF systems has been the use of electrokinetic space–charge model (ESCM) that describe the creeping flow of charged species through charged capillaries. In the ESCM approach, ions are treated as point charges, the radial distribution of which across the pores of the NF is defined by the Poisson–Boltzmann equation and the ion transport along the pore being described by the extended Nernst–Planck equation [142]. However, the application of these models is limited by the numerical complexity of calculations [143]. Bowen et al. [144] proposed Donnan-Steric Pore Model (DSPM); where ion transport is described by the extended Nernst–Planck equation, modified to include hindered transport, with equilibrium partitioning due to a combination of electrical (Donnan) and sieving (steric) mechanisms. In the DSPM model, NF performance is analyzed as a function of volumetric flux and is dependent on three parameters, respectively: effective pore radius, r_p_; effective ratio of membrane thickness to porosity, ∆x/A_k_; and effective membrane charge density, X_d._ The model has been successfully used for prediction of performance of NF for uncharged solutes and monovalent salts [145]. However, the model exhibits poor quality of agreement with experimental data in case of multivalent cations such as Mg^2+^ [146]. There are many variations on this basic modelling theme in existence; in essence they are all intrinsically related to the Nernst–Planck equation. The DSPM model has been further developed more rigorously to include the dependence of chemical potential on pressure on solute transport, an increased solvent viscosity within the pores and dielectric exclusion. The effects of pressure on chemical potential, and hence on solute transport, and of a pore radius dependent viscosity are included [143]. There has been much debate over the nature of this effect and more rigorous description to capture the performance of NF in a wide range of practical situations and this has been summarized in a recent reviews [135,139].

### 4.4. NF Membranes for Arsenic Removal

Remarkable progress in new materials and methods in membrane technology has created opportunity to produce nanofiltration (NF) membranes with higher selectivity and increased water flux at a much lower operating pressure. Modern NF technology enables a wide range of water resource pollutants to be controlled and managed efficiently. Separation of ionic species by an NF membrane strongly depends on the membrane properties (membrane charge and membrane pore radius and pore distribution). A membrane with smaller pores is better able to retain ionic species but needs higher energy requirement. Likewise, a highly charged membrane is better able to exclude co-ions (ions of like charge as the membrane) from the membrane structure. Therefore, knowledge and understanding of the characteristics of the variety of the NF membranes and their performance can allow for a priori predictions of the separation behavior of the membrane. The following sections discuss in some detail the different types of NF membranes that are either available commercially and at an early stage of development.

#### 4.4.1. Commercial NF Membranes for As Removal

Nanofiltration (NF) membranes have a dramatic development since they were first introduced in the late 1980s; and in 1993/1994, NF membranes were employed for As removal [147,148]. In these early investigations, thin film composite (TFC) NF membranes were employed that were able to remove up to 97 percent of As^V^. Since then, researchers have used commercial NF membranes of different kinds and qualities from different manufacturers, including: NF-45, NF70 4040-B, HL-4040F1550, 4040-UHA-ESNA, ES-10, NTR- 7250, NTR-729HF, BQ01, NTR-729HF, NF-90, NF-200, NE 90, NF 300. The studies of As removal by nanofiltration process at various operational conditions that were carried out so far are presented in Table 4 with the observed rejection percentage of arsenate and arsenite, although most of the studies were done on arsenate only.

#### 4.4.2. Synthesized and Modified NF Membranes for As Removal

The performance of commercially available thin film composite nanofiltration (NF) membranes reported so far has significant room for further improvement. Therefore, besides commercially available NF membranes, various membrane fabrication and innovative modification techniques have been developed and employed in order to improve the performance and overcome the drawbacks (Table 4). The most desirable improvements for NF include: (a) increase in separation, rejection efficiency and permeance; (b) reduction in membrane fouling; (c) improvement in membrane lifetime and chemical resistance; and (d) reduction in cost. Interfacial polymerization (IP) and grafting polymerization (GP) have been identified as the two most practical and useful techniques for NF modification. In general, current state-of-the-art NF membranes are based on thin film nanocomposite-NF (TFC-NF), which is formed by deposition of an active layer (e.g., polyamide, PA, USA), on top of a porous support layer that is typically an ultrafiltration (UF) or a microfiltration (MF) membrane. Most often, the active layer on suitable support membrane is formed by a simple IP and GP technique. The IP method typically involves phase inversion followed by interfacial polymerization to produce TFC membranes. The performance of TFC-NF membrane, in terms of permeance and selectivity is primarily determined by the active layer. Such TFC structures deliver high performance membranes with strong mechanical integrity at low cost with tunability and scalability [171].

**Figure 9:**
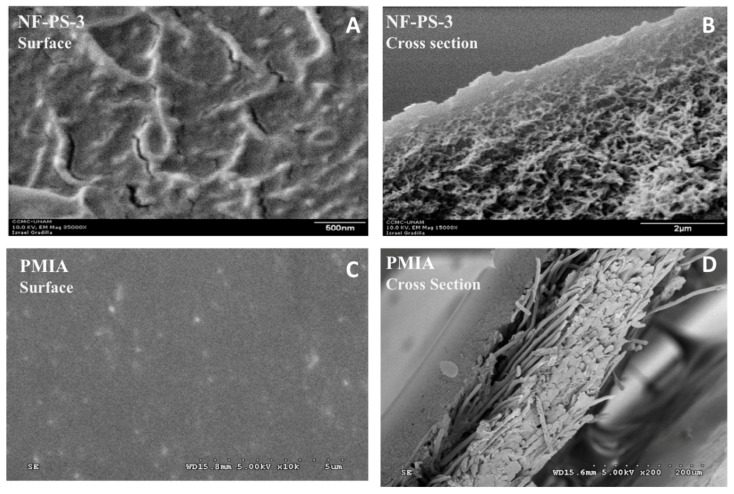
Scanning electron micrograph (SEM) images of surface morphology and cross-sectional structure of typical TFC-NF membranes: (**A**) surface image (at 35,000× and 500 nm scale); (**B**) cross-sectional image (at 35,000× and 2 μm scale) of NF-PS-3 membrane; (**C**) surface 10,000×; and (**D**) cross-sectional 2000× views of a typical PMIA asymmetric membrane. (adapted with permission from [163,164,166,167,168]. (PMIA = Poly m–phenylene isophthalamide)

To enhance As removal, the negatively charged polyamide nanofiltration membrane NF-PS-3 (Table 4) was synthesized by Pérez-Sicairos et al., using the combination of phase inversion and interfacial polymerization process [163]. The synthesis of this membrane was performed in two steps; firstly, phase inversion process was used to prepare the microporous polysulfone membrane and then the polyamide thin-film was coated on the microporous polysulfone substrate using IP process of a secondary amine in aqueous phase with an acid chloride in organic phase. The surface and cross-sectional views, SEM micrographs, of a typical NF-PS-3 membrane are shown in Figure 9A,B. NF-PS-3 membrane was prepared via interfacial polymerizing process between an aqueous secondary amine solution containing 0.25% *w*/*w* piperazine, 0.25% *w*/*w* polyvinyl alcohol, 0.5% *w*/*w* sodium hydroxide, and a hexane solution containing 1.0% *w*/*w* trimesoyl chloride (TMC) (156). The synthesized TFC-NF membrane was able to remove both arsenate and arsenite to a higher level than the commercial NF membranes, which was 98.5 and 70.4% respectively (Table 4). Besides the effect of the negative surface charge of the membrane, it has been observed that applied potential increased the As^V^ rejection by 48.2% when it was increased from 0 to 2.0 V for a feed containing 1000 ppb. For the same change of applied potential, rejection of As^III^ was increased from 52.3 to 70.4%. Zhao et al. developed a PMIA (Poly m–phenylene isophthalamide) asymmetric nanofiltration membrane and reported more than 90% rejection of arsenate over a wide range of As feed concentration (Table 4) [157]. This phenomenon could be attributed to the higher rigidity of the membrane as well as to the high surface free energy of PMIA that caused the hydrophilicity. It was reported that the presence of NaCl enhanced As^V^ rejection and this rejection could reach up to 99% at a feed As concentration of 100 μg/L, whereas there was a rejection decrease of 8% in the presence of Na_2_SO_4_. SEM micrographs of Figure 9C,D illustrates the surface and cross-sectional views of a typical PMIA asymmetric membrane. The dense and tight surface structure of PMIA membrane is clearly visible from Figure 9C. From Figure 9D, the cross-section of the NF membrane, it is evident that the originally porous surface of substrate is covered by a flat featureless PMIA layer.

**Figure 10:**
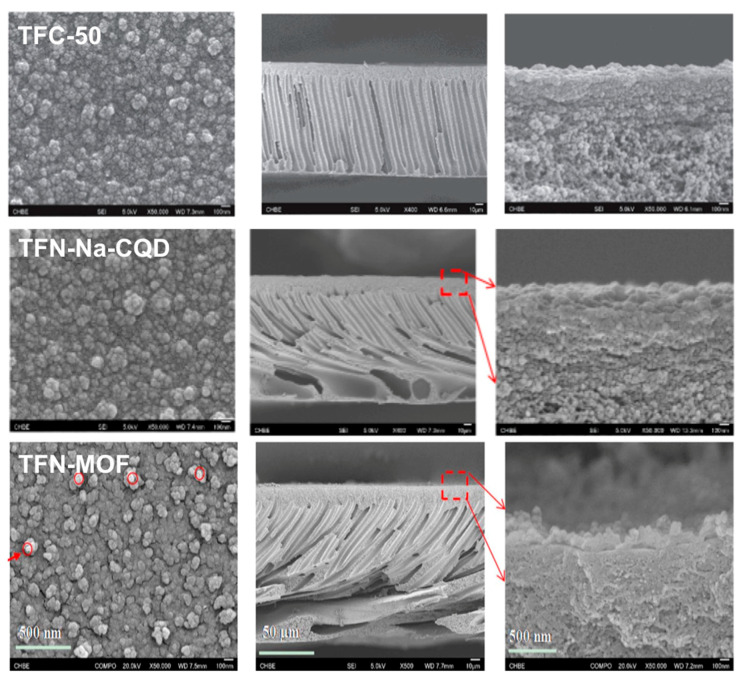
SEM micrographs of surface and cross sectional morphology of synthesized membranes: TFC-50-P[MPC-co-AEMA] co-polymer modified thin-film composite (TFC) membrane with 50 wt% loading of P[MPC-co-AEMA] (adapted with permission from [163,164,166,167,168]); TFN-Na-CQD- sodium ion modified carbon quantum dot (Na-CQD) incorporated thin-film nanocomposite (TFN) membranes with Na-CQD loadings of 0.05 wt% (adapted with permission from [163,164,166,167,168]); TFN-MOF- thin-film nanocomposite membranes modified with water stable zirconium metal-organic framework (MOF) UiO-66 nanoparticles with diameters of 30 nm (adapted with permission from [163,164,166,167,168]).

An Impressive rejection of As^V^ (99.8%) was obtained from the NF composite membrane, TFC-50 (Table 4), where the TFC membranes were molecularly designed and synthesized by incorporating a zwitterionic co-polymer P[MPC-co-AEMA] during the interfacial polymerization of PIP and TMC [168]. To achieve the goal, as a first step a water-soluble zwitterionic co-polymer, P[MPC-co-AEMA] was synthesized vis a single-step free-radical polymerization between 2-methacryloyloxyethyl phosphorylcholine (MPC) and 2-aminoethyl methacrylate hydrochloride (AEMA). TFC-50 membranes demonstrated improved pure water permeability (PWP) and impressive rejections rate of 98.2%, 99.1%, and 99.8% toward SeO_3_^2−^, SeO_4_^2−^, and HAsO_4_^2−^, respectively. Higher rejections are also obtained in the simultaneous removal of Se and As. SEM micrographs of the surface and cross-sectional morphology of the TFC-NF membranes depicts the unique surface and interfacial morphology along with pore quality (Figure 10: TFC-50). Polyamide intercalated TFC-NF membrane modified with biofunctionalized core shell composite (PA-CSBF) membranes showed improved pure water permeability and rejections for As^III^ and Se ions due to their enhanced hydrophilicity. An optimum PA-CSBF membrane exhibits 99 and 98% rejection of As^III^ and Se ions, at permeate flux of 444 L/m^2^h, (Table 4), respectively [169]. Fully aromatic polyester TFC-NF membranes were successfully prepared on polysulfone support through interfacial polymerization (IP) of trimesoyl chloride (TMC) with bi functional resorcinol (Res), tri-functional phloroglucinol (Phg) and their blends for effective arsenic removal. These membranes are negatively charged under operating pH conditions, as a result, divalent anions were efficiently rejected. The membranes showed promise for arsenic removal, giving 70–90% rejection of Na_2_HAsO_4_ depending on the ionic strength and pH of the feed water. The active layer thickness and pore radius can be tuned through controlling the amount of Phg. The functionality of phenol greatly influenced the properties and performances of the membranes [166]. Song et al. [172] prepared a TFC hollow NF membrane via the solution coating process. The hollow UF fiber was a commercial PES membrane. The amorphous polyether ether ketone was sulfonated and dissolved in methanol. In a successive step the solution was filtered and coated on the inner surface of the hollow fiber membrane. The obtained membrane achieved >95% rejection of As^V^ (Table 4) and water flux of 11 Lm^−2^h^−1^bar^−1^ [165]. 

He et al. [167] attempted to develop a novel thin film nanocomposite (TFN) NF membrane through further modification of TMC-NF membrane using a facile technique by loading Na^+^ functionalized quantum dots (Na-CQDs) in the PIP solution prior to the interfacial polymerization of PIP and TMC. It was reported that Na-CQDs can be easily dispersed in the PA-selective layer to efficiently manipulate the surface structure and hydrophilicity of the membranes. The surface and cross-sectional morphology of the synthesized TFN-NF membranes is shown in Figure 10 (TFN-NaCQD). The tailored membrane showed enhanced flux and rejection of As^V^ because of the higher hydrophilicity of the membrane [167] [see Table 4, TFN-0.05]. A similar TFN-NF membrane was also prepared where the doping particle used was water stable zirconium metal-organic framework (MOF) UiO-66 nanoparticles with different diameters, e.g., 30, 100, and 500 nm [160]. The TFN membrane comprising 30 nm UiO-66 exhibited the best performance among the three TFN-NF membranes. These membranes were tailored to remove both Se and As concurrently. The surface and cross-sectional morphology of the synthesized TFN with 30 nm UiO-66 are shown in SEM micrograph of Figure 10 (TFN-MOF). Compared to the thin-film composite-TFC membranes, the TFN membranes exhibit higher pure water permeability (PWP) and rejections for both pollutants due to their smaller pore size and higher hydrophilicity. The TFN membrane exhibits water flux of 11.5 Lm^−2^h^−1^bar^−1^, achieved 98.6% rejection of As^V^, and enhances robustness [see Table 4, TFN]. Novel thin film nanocomposite- TFN-NF membrane via the interfacial polymerization method incorporating polyhedral oligomeric silsesquioxane (POSS) with various functional groups as a modifying nanomaterial has also been examined [173]. In the process, PES substrate was first prepared by phase inversion method; subsequently, the selective layer was fixed on the PES substrate by interfacial polymerization between Piperazine (PIP) and 1,3,5-benzenetricarbonyltrichloride (TMC). Initially, the PES substrate was immersed in PIP solution and dried. Then TMC/hexane solution was poured on the dried membrane and dried again in the air. To make POSS-TFN NF membranes POSS with different functional groups including: octaammonium POSS (P-8NH_3_Cl), PEG POSS cage mixture (P-8PEG), and OctaPhenyl POSS (P-8Phenyl) were incorporated within NF. Depending on the hydrophilicity or hydrophobicity, POSS was incorporated into the polyamide layer in two ways; either adding hydrophobic POSS in the organic phase or introducing hydrophilic POSS in the aqueous phase. The resultant NF membrane has a mean effective pore diameter of 0.65 nm, a molecular weight cut off of 302 Da and pure water permeability of 5.4 LMH/bar, with impressive high rejections of HAsO_4_^2−^ to 97.4%. A TFN-NF membrane incorporated with aromatic amine functionalized multiwalled carbon nanotubes (AAF-NF); where polyamide layer was synthesized by IP between piperazine and trimesoyl chloride monomers showed ability to efficient removal of As^V^ from polluted groundwater and long-lifetimes. Water permeate flux and the arsenic rejection of the AAF–NF membrane increased by 15% when it is compared with a typical commercial semi-aromatic polyamide nanofiltration membranes [170]. A variety of methods, specifically the use of an innovative nanotechnology approach, have recently been developed to improve the performance of NF, and are summarized as follows in Section 5.

## 5. Nanofiltration Membrane Fabrication Process: Limitations and Future Prospects

Different nanofiltration membrane fabrication method was developed focusing on the resulting pore size (1–10 micron) of the membrane. The most common fabrication method for making polymeric nanofiltration membranes is interfacial polymerization. It also discussed in the previous section that the interfacial polymerization method has been used to make different types of NF membrane for arsenic removal. In this process, an ultrafiltration membrane substrate is used to support the selective layer of the membrane. It is formed in a TFC structure. The thin layer is formed by a reaction between two reactive monomers. Several different types of reactive monomers/prepolymers including bisphenol-A (BPA), tannic acid, m-phenylenediamine (MPD), diethylenetriamine (DETA), triethylenetetramine (TETA), tetraethylenepentamine (TEPA), piperazidine (PIP), polyvinylamine reacting with trimesoyl chloride (TMC), or isophthaloyl chloride have been successfully employed using IP process to form the thin active film layer in TFC-NF [174,175,176,177]. Polyhexamethylene guanidine hydrochloride (PHGH) has been successfully used as a monomer to form the active layer using the IP method to prepare NF membrane with excellent bacteria inhibition characteristics [178]. Wang et al., reported a simple elegant method for the fabrication of an efficient TFC-NF membrane with a crumpled polyamide (PA) layer via IF on a single-walled carbon nanotubes/polyether sulfone composite support membrane loaded with sacrificial nanoparticles [179]. Recently, a novel approach to design TFC membranes with ultrahigh permeance using hydrogel assisted IF has been advanced, which is facile, and enables cost-efficient and scalable manufacturing [180]. 

Regardless of their advantages, only a limited number of polymeric NF membranes can be prepared using interfacial polymerization, which limits several key improvements of the membrane such as hydrophilicity, antifouling property, chemical resistance, longer lifespan, and rejection efficiency. Though, incorporation of polymer in the membrane/polymer solution prior to the interfacial reaction often helps to improve the property of the membrane. The recent progress in the NF membrane largely focuses on fabricating the thin-film nanocomposite (TFN) membrane by adding nanoparticles [135]. TFN-NF incorporating additives such as nanoparticles in the active layer has also been promoted. TFC-NF membranes using IP technique and based on polyetherimide (PEI) modified with amine-functionalized silica NPs has been employed to improve the mechanical and thermal stability of the membranes [181]. The introduction of inorganic salt (e.g., calcium chloride (CaCl_2_) dissolved in aqueous phase and organic acids with different structures such as ascorbic acid, citric acid, malic acid, and acidic strengths were studied during the fabrication and used as an additive during the IP process [182,183]. Metal-alkoxide (e.g., titanium tetraisopropoxide, bis-(triethoxysilyl)-ethane phenyltriethoxysilane) assisted IP has also been reported for the synthesis of inorganic-polyamide nanocomposite membranes to improve the permeability performance [184]. NF membranes, consisting of a composite barrier layer prepared by interfacial polymerization of polyamide around the ultra-fine cellulose nanofibers (CN) layer in a thin-film nanofibrous composite (TFNC) scaffold were demonstrated [185]. However, there are some problems associated with the incorporation of nanoparticle in the IP membrane such as poor dispersion and agglomeration of particles. A variety of surface modifications [186], zwitterionization [187,188], and hybridization [189] during IF process have been employed to enhance the performance of NFs. 

The NF membrane is preferred to be very thin for getting high water flux. The porous support layer is the unavoidable part of the TFC NF membrane to support the thin selective upper layer. The support layer is prepared using the phase inversion process. Therefore, it increases the steps in the membrane fabrication process. Besides, usually a very toxic solvent is used in the phase inversion process to make the ultrafiltration membrane support. To avoid some of the critical disadvantages, electrospun membrane has been developed to replace the conventional membrane support system; as the transport properties of the membrane is enhanced by interconnected pore structure, which permits a shorter path for water transport [190,191]. In addition, the electrospun nanofiber membrane can be thin with good mechanical properties [192] as discussed below.

### 5.1. Electrospinning: An Emerging Synthesis Technique of NF Membranes

**Figure 11:**
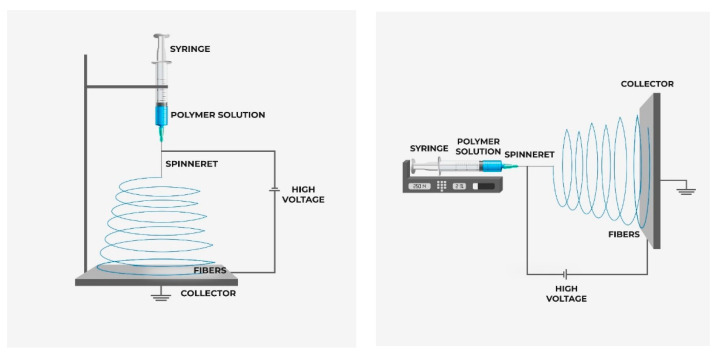
Schematic diagram of electrospinning process (vertical and horizontal).

Electrospinning is a flexible technique for making nanofibrous porous membranes for different applications such as wastewater treatment, desalination, and filtration [193,194]. Several advantages like low start-up cost, high surface area to volume ratio, 3D interconnected pore structure, more contacting surface (the fiber surface), high strength, and feasibility in making well dispersed mixed matrix membrane make it a viable fabrication technique for membrane preparation. In addition, the electrospun membrane can be prepared from most of the major classes of the polymer [195]. The polymer is taken in solution and the filler materials like nanoparticle are dispersed to the polymer solution prior to the electrospinning and then the polymer solution is loaded in the syringe and placed in the syringe pump as shown in Figure 11. A high voltage is applied between the stationary or rotating collector and the spinneret to make the fiber from the jet solution. The nanofiber is accumulated in the collector to make the nanofibrous membrane [196]. The porosity of the membrane depends on the operational parameters such as voltage, feed rate, the distance between collector and spinneret, operation time, and solution property. By tailoring the solution, property and operational parameters desired membrane morphology can be obtained. There is room for more research to make a versatile NF membrane using electrospinning technology.

### 5.2. Graphene-Based Materials for Arsenic Removal

**Figure 12:**
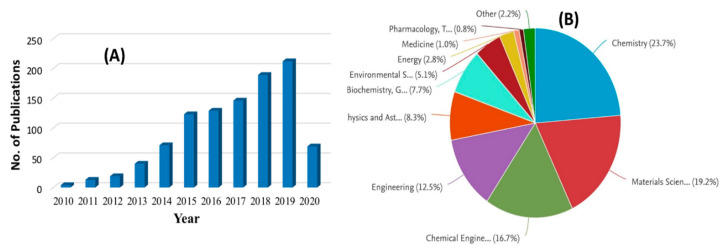
(**A**) The number of publications and (**B**) application of graphene oxide (GO) in various fields based on the keyword “graphene oxide nanocomposite membranes” in Scopus database (data collected on 14.04.2020).

Graphene oxide (GO) has different functional groups (hydroxyl, carboxyl, epoxide, and C=C) on the surface, which makes it a preferred and desired nanomaterial to use solely and as a filler material in different types of membranes for the modification and enhancing the properties of the membrane. The addition of GO in the membrane enhances the water molecules transportation through the membrane by forming the interconnected nanochannels [197]. GO is also hydrophilic in nature due to the presence of the hydrophilic group as well as has great antifouling, antibiofouling, and antibacterial properties [198,199,200]. The wide attention paid to GO is shown in Figure 12 as well as the application in various fields. GO has been used solely as the adsorbents for the removal of arsenic, where the efficiency has been found 100% for arsenate of a 20-mg/L concentration [201]. In the same research, 100% arsenate removal was also achieved by a nanocomposite of GO and 3-aminopyrazole. Mostly, the graphene-based materials used as nanocomposites are consist of metal oxides or organic/inorganic compounds with GO or reduced graphene oxide (rGO). Iron oxides (mostly Fe_3_O_4_), copper oxides, etc. were used with GO or rGO in the nanocomposites [202,203,204,205,206], some organic compounds such as epoxy, aminopyrazole, EDTA-Chitosan, Imino-Thiobiuret, etc. [201,207,208,209] and inorganic compounds like LD-Hydroxide, silica, etc. [210,211] have been used with GO or rGO to prepare the nanocomposites as well. The arsenate removal efficiency of these nanocomposites is in the range of 80–100%. The hydrothermal treatment, solid state dispersion, and aqueous thermal treatment have been used for the synthesis of these nanocomposites.

## 6. Influential Parameters on Arsenic Removal Efficiency

The effects of different operational conditions like applied pressure, feedwater temperature, and the solution chemical composition such as As oxidation state and level, pH, and presence of co-occurring inorganic solutes was studied in detail by Waypa et al. in 1997 for the first time [121]. Due to their practical importance, the effects of different parameters have been evaluated by many investigators using both synthetic freshwater and source water utilizing bench-scale experiments. The effect of the operational parameters on the rejection of both arsenite and arsenate by NF membranes is summarized in Table 5.

### 6.1. Effect of Arsenic Concentration of Feed Water

Concentration of As^V^ itself effects the removal efficiency of As^V^ as the removal of As^V^ decreased dramatically with the recovery (percent of feedwater converted to product water) of the system increased [147,148], due to the dominant role of charge exclusion in the removal of arsenate [212]. This increasing trend of arsenate rejection is not proportional to the retentate concentration [213]. On the other hand, the increased arsenic concentration in feed water decreases As^III^ removal efficiency [212], as both the diffusion and convection of the uncharged arsenite increases with the increase of arsenic concentration in feed water, which results in lower arsenite rejection [213].

### 6.2. Effect of Co-Occurring Inorganic Solutes on As Removal

Some ions such as Na^+^, Ca^2+^, etc. are present in the surface water and ground water, which also influences the arsenic removal efficiency. Presence of calcium ion increases the removal of both arsenate and arsenite as Ca^2+^ interacts with the membrane and reduces the negative charge of the membrane [221], which results in less electrostatic repulsion between the negatively charged sites within the membrane structure. Therefore, the pores of the membrane are compressed in a narrower size, which blocks the arsenic ions more and results in high removal of arsenic. On the other hand, existence of Na^+^ ions as NaCl salt decreases the arsenate rejection as the presence of salts in the solution weakens the charge exclusion and the dominant rejection mechanism of arsenic rejection process is the Donnan (charge) exclusion [212]. Furthermore, sulphate (SO_4_^2−^) and phosphate (PO_4_^2−^) doesn’t have a remarkable effect on both the arsenate and arsenite rejection, as the permeabilities of these species are comparable to As^V^ species and the uncharged As^III^ species did not interact with the negatively charged sulfate or phosphate species [121].

### 6.3. Effect of Feed Water pH 

Existing form of arsenic in water changes with the pH of water, which influences the arsenic removal significantly. Arsenite is the neutral form of arsenic that remains unchanged up to pH 8, when arsenate remains as an anion. According to Donnan exclusion theory, arsenic rejection is affected by the charge valence of arsenate in medium due to the negative charge of nanofiltration membrane. Arsenate changes from its monovalent form (H_2_AsO_4_^−^) to divalent form (HAsO_4_^2−^) with pH and this larger hydrated radii of divalent ion compared to monovalent ions is the another possible cause for increased rejection of arsenate species [162]. With the change of pH, the pore size of NF membrane also changes. At the pore surface point of zero charge (isoelectric point), the membrane functional groups are minimal in charge and hence open up, as the absence of repulsion forces contribute to the widening of the membrane pores. At high or low pH values, functional groups of membrane polymer can dissociate and take on positive or negative charge functions. Repulsion between these functions in the membrane polymer reduces membrane pores.

### 6.4. Effect of Applied Pressure

Increasing the applied pressure during nanofiltration process increase the arsenic removal efficiency, as the permeate flux increases with increased applied pressure (according to Equation (1)) with lower arsenic concentration due to the dilution effect [121].

*J_v_* = *A* (Δ*Ρ* − *σ*Δ*n*)………. (1)

This is a steady-state transport equation of water [222], where *J_v_* is the water volumetric flux, *A* is the water permeation coefficient, *σ* is the reflection coefficient, *P* is the hydraulic pressure, and *n* is the osmotic pressure. In Equation (1), Δ*Ρ* = *P_f_* − *P_p_* and Δ*n* = *n_w_* − *n_p_*, here the subscripts *f*, *p*, and *w* refer to the high-pressure side of the membrane (feed water), the low-pressure side of the membrane (permeate water), and the membrane surface, respectively. Removal of arsenic increases with the increase of the applied pressure, but it does not follow any specific trend with the increase of the same amount of pressure. With the increase of 0.8 MPa pressure, removal of arsenate increases by 8.5% in the case of NTR-7450, but 4.2% for UTC-70 [151] and the arsenite removal was increased by 8 and 10.5%, respectively, as presented in Table 5.

### 6.5. Effect of Temperature

Rejection of arsenic decreases with increasing temperature as the diffusivity of arsenic increases with temperature, which results in increased diffusive transport of arsenic through the membrane [219]. The trend of arsenic removal with the change of temperature is not the same for all membranes as the applied pressure, such as increasing by 20 °C in temperature, the arsenate removal decreases 1.3% for NF-90 membrane, but 1.7% for NF-200 [219]. Besides this, the changing trend of arsenate and arsenite removal is the same, as a 5% decrease in the removal efficiency for both in the case of NF90-4040 with the increase of 9 °C temperature [160].

## 7. Current Challenges and Limitations

Although NF membranes offer several unique properties for application in the arsenic removal technology, large scale application is still in the premature stage because of several obstacles such as lack of research on membrane materials, low durability of membranes, and water flux declination with time. Most of the research groups focused on the application of commercially available NF membranes rather than synthesizing new membrane specialized on arsenic removal. Though arsenite is more toxic than arsenate, most of the research reports were focused on the arsenate removal as the removal process is easier due to the Donnan exclusion effect of membranes. Most of the current state-of-the-art commercial membrane technology for NF is based on a cross-linked polyamide membrane chemistry. The most commonly employed amines are piperazine, while trimesoyl chloride (TMC) is the most common acid chloride, leading to a cross-linked three-dimensional structure with residual end-groups as amine and COOH. In TFC, the barrier layer has a thickness of ca. 200 nm, and is generally an integral part of a three-layered composite membrane. In such a three-layered composite membrane, typically the bottom layer is a polyethylene terephthalate (PET) reinforcing web to provide mechanical support, followed by an asymmetric polysulfone (PS) layer with surface porosity in the tens of nanometers, and finally the polyamide barrier layer for AS removal. NF polyamides are negatively charged at practical operating conditions, a product of the balance between dissociated free acid and amine end groups. These negatively-charged membrane surfaces are more susceptible to membrane fouling activity. The swelling tendency and lower mechanical strength of the membranes also decrease the durability of NF membranes for As removal as well as the membrane fouling decreases the water flux with time. Although, the mixed matrix membrane and thin film nanocomposites could be the solution to the existing problems, extensive research is required to establish a mixed matrix membrane for efficient arsenic removal. In addition, a NF membrane synthesis process for As removal has not been explored yet, instead, mostly interfacial polymerization has been used to prepare the NF membranes for arsenic removal. Another hurdle is the reduced possibility of ensuring suitable pore size and distribution in large-scale production.

## 8. Conclusions and Outlook

In recent years, approaches for the establishment of a membrane-based arsenic removal technique is a highly active research field. Among various membrane types, the NF membranes are leading because of their impressive arsenic rejection with high flux and low-pressure operation. In this review, recent developments of the NF membranes for arsenic removal have been discussed and our comparative analysis reveals several insights for their structure-property-performance relationship and their practical implementation. It appears that the current studies mostly focused on commercial TFC membranes. The commercial NF membranes, however, are not effective for the successful removal of both As^III^ and As^V^. Moreover, arsenic rejection and water flux have not been studied extensively. Although new advanced NF membranes including thin film nanocomposite (TFN)/mix-matrix NF membranes are emerging and being gradually introduced for arsenic removal; however, the research is still at a premature level. Future research should focus on introducing new membrane material, novel low cost, an environmentally benign membrane synthesis process to fabricate advanced TFC/TFN membranes in order to improve the water flux, rejection of both As^V^ and As^III^, and decrease the membrane fouling. By focusing on all the mentioned features, NF membranes have the potential to be the most efficient and economic arsenic removal method and it would be possible to adopt it for water treatment in the affected areas, both at a household and community level. 

An exciting number of next-generation polymer materials are currently being investigated that have the potential to overcome the current limitations and supersede the energy and separation capabilities of industry standard polyamides NFs. Among them, block copolymers (BCP) and liquid crystals (LC) membranes have demonstrated a remarkable ability as NF membranes [223]. BCP is one of the most versatile and tailorable materials. Not only their chemical compositions can be tailored over a wide range of composition levels, but also varying processing criterion such as the solvent interactions can also be utilized as a tool to tailor NF membrane porosity and morphology. Moreover, in BCP-based NFs, additional functionalities such as antifouling properties [224], pH responsiveness [225], and thermal responsiveness [226] could be easily introduced using post-processing steps. In such stimuli responses of NFs membranes, the flux of the membranes could be altered by both pH and temperature. The flux in LC membranes are reported to be orders of magnitude below those of BCP and polyamide membranes [223]. In the LC space, the potential of using polymerizable lyotropic liquid crystal (LLC) assemblies as NF membranes has been demonstrated [227]. The biggest challenge facing BCP and LC self-assembled membranes is the cost of materials, compared to conventional materials used for NF applications. The processability of these systems, particularly BCPs are readily tailorable to large-scale manufacturing and with appropriate investment in scale-up technologies the gap could be overcome in the future for wider applications.

Recently, 2D materials, specifically graphene, have attracted significant attention as potential NF membrane material because they hold a promise of providing minimum resistance to water transport. Graphene, by definition is only a single atomic layer thick, and is a truly 2D material. Computational studies have demonstrated high predicted rates of water transport and salt rejection through nanoporous graphene membranes [228]. The aromatic rings of the honeycomb lattice of graphene are too small for the passage of molecules such as salts, water, and even gases. Thus, creation of intentional nanopores of requested size and uniform distribution for water transport has been developed as a potential approach to make an effective NF membrane [229]. Graphene sheets can be transformed into membranes in a variety of ways: (i) composite blends with polymers or other matrix structures, (ii) stacks of sheets, or (iii) truly 2D single layer or few layer continuous membranes [223]. Though considered ideal, graphene presents greater challenges in (i) large area scalability, (ii) generation of monodisperse distribution of optimally sized pores, (iii) the complexity of processing, and (iv) cost of production. Pore uniformity and defect management are the two major factors limiting the accelerated development of nanoporous graphene membranes, even for high-end applications. To mitigate such problems, graphene oxide (GO) membranes formed in layer-by-layer (LBL) GO structures that behaved quite similar to commercial NF, have been attempted successfully. GO flakes can be prepared by chemical oxidation of abundantly available and sustainable graphite followed by exfoliation to release individual or few layer nanosheets into aqueous suspension [230,231]. The sheets can range in size from a few hundred nanometers up to a few micrometers. Film formation from such dispersions becomes much more straightforward, and consequently, GO membranes used in water filtration are generally composed of layered GO flakes coated onto a microporous support surface. The flow profile in a GO laminate film is one of percolation, with lateral flow along hydrophobic channels combined with periodic perpendicular transport through spacing between adjacent nanosheets, as well as holes of imperfection within sheets. While many GO membranes have provided ultrahigh water permeability, their selectivity for ionic solutes, thus far, have not been competitive against current NF membranes, which may result from defects introduced during the casting of the thin films.

Biomimetic membrane technology-drawing inspiration from nature’s own ways of transporting and purifying water has recently been a motivation for development of more efficient separation membranes to purify water faster and more efficiently than ever before. Over millions of years, nature has evolved remarkable water channel proteins, e.g., aquaporins, which are crucial for life in all organisms. They facilitate rapid, highly selective water transport across the cell membrane [232]. These channels are so important that in 2003, the Nobel Prize in chemistry was awarded to Professor Peter Agre for his discovery of the aquaporin water channel. Aquaporin-embedded biomimetic membranes for nanofiltration have recently been conceptualized and fabricated, and can give an impressive water permeability and salt rejection [233,234]. These studies open up new possibilities of aquaporin embedded biomimetic membranes for water purification with advantages that include high throughput with less energy consumption. Aquaporin-based biomimetic NF membranes for suppliers of low-pressure household water purifiers and membranes for NASA to recycle astronaut urine in space has been developed on a commercial scale [235]. Although these artificial biomimetic systems hold tremendous promise by combining the functionality of biological channels with facile processability of synthetic materials, they are still in the nascent research phase with significant scale-up challenge. The drawback of the use of proteins in non-natural environments is that their life time may be limited by their stability and degradation. However, the major driver for development, such as biomimetic membrane technology, is that the conventional membrane technologies have reached their performance limit and new technologies with higher productivity and efficiency are essential.

Finally, the total global NF market is driven by its applications and demand from end user sectors, including water and wastewater treatment, food and beverages, chemical and petrochemicals, pharmaceutical and biomedical, textile and metalworking industry. Nanofiltration is highly adopted, growing technology and in water treatment NF are used not only for As removal but also water softening, color removal, as a barrier for removing various viruses and bacteria, industrial waste water treatment, water reuse, and even desalination. The increase in the use of chemical free water treatment procedures across various industries provide significant potential growth opportunities for the NF market in the future. The growth of the nanofiltration membrane market is expected to accelerate due to rapid urbanization and industrialization across emerging economies such as India and China; and increase in demand for water for domestic and industrial purposes, access to fresh and clean water. The global nanofiltration membrane market was valued at $643.22 million in 2017, and is projected to reach $954.65 million by 2025, growing at a compound annual growth rate (CAGR) of 5.4% from 2018 to 2025 [236]. However, high installation costs and lack of funds in the emerging economies may restrict the market growth.

## Figures and Tables

**Table 1:** Arsenic (As) contamination in natural groundwater in various countries (adapted with permission from [37]).

Serial Number	Country	Region	Groundwater As Level (ppb) *	Permissible Limit (ppb)	References
1	Afghanistan	Ghazni	10–500	10 (WHO)	[39]
2	Australia	Victoria (around the gold-mining regions)	1–12 (Groundwater); 1–73 (Drinking-water); 1–220 (Surface water)	--	[39,40,41]
3	Bangladesh	Noakhali	<1–4730	50 (WHO)	[39,42,43]
4	Brazil	Minas Gerais (Southeastern Brazil)	0.4–350 (Surface water)	10 (WHO)	[40,41,44]
5	Cambodia	Prey Veng and Kandal-Mekong delta	Up to 9001–1610	10 (WHO)	[39,45]
6	Canada	Nova Scotia (Halifax county)	1.5–738.8	10 (WHO)	[39,40]
7	China	--	50–4440	50 (WHO)	[46]
8	Finland	Southwest Finland	17–980	10 (WHO)	[40,41,44]
9	Greece	Fairbanks (mine tailings)	Up to 10,000	10 (WHO)	[36,39]
10	India	West Bengal & Uttar Pradesh	10–3200	50 (WHO)	[36,39,41,47,48]
11	Japan	Fukuoka Prefecture (southern region)	1–293	10 (WHO)	[40,41]
12	Mexico	Lagunera	8–620	25	[36,39,41]
13	Nepal	Rupandehi	Up to 2620	50	[39,46]
14	Pakistan	Muzaffargarh (southwestern Punjab)	Up to 906	50	[40,41,49]
15	Taiwan	--	10–1820	10 (WHO)	[36,39,41]
16	Thailand	Ron Phibun	1–>5000	10 (WHO)	[36,39,41]
17	USA	Tulare Lake	Up to 2600	10 (USEPA)	[39,50,51]
18	Vietnam	Red River Delta (Northern Vietnam) Mekong Delta (Southern Vietnam)	<1–3050	10 (WHO)	[36,46]

* ppb = parts per billion.

**Table 2:** Arsenic contamination in the surface water area.

Surface Water Area	Arsenic Contamination (µg L^−1^)	Ref.
Stampede and Slate Creek watersheds of USA and Alaska	239	[52]
Manchar Lake, Pakistan	60.45	[53]
Zenne River, Belgium	0.97–3.6	[54]
Alpine/Mediterranean Var River, France	0.1–263	[55]
Gomati river (Ganga Plain, northern India)	1.29–9.62	[56]

**Table 3:** Advantages and disadvantages of various arsenic removal technologies.

Arsenic Removal Technology	Advantages	Disadvantages	Removal Efficiency of As^(v)^ (%)	References
Oxidation	-Low operating cost. -Works over a wide pH range.	-Very slow process. -Drinking water has bad smell and color in addition of chlorine, permanganate, etc. -Sludge formation.	>95	[39,68,75,76,77,78,79,80,81,82,83,84,85,86,87,88]
Coagulation	-It can be operated within a wide range of pH.	-Pre-oxidation of arsenite required. -High arsenic contaminated sludge production. -Expensive process. -Additional filtration required.	>90	[84,89,90,91,92,93,94,95]
Adsorption	-Low cost. -Ease of operation.	-pH, effective surface area, and the nature of the adsorbent need to be maintained. -Arsenite cannot be removed very well. -Post-filtration required. -Organic matter, other salts in water decreases the efficiency of the process. -Removal of the generated heavy flocs are difficult.	100	[24,94,96,97,98,99,100,101,102,103,104,105,106,107]
Ion Exchange Process	-pH independent process. -Only efficient for the arsenite removal.	-Only efficient for the arsenite removal. -Expensive process. -Low capacity. -Sludge disposal problem. -Resin needs to be replaced again and again.	95	[94,106,108]
Electrocoagulation	-Less area requirement. -Sustainable technology.	-Sludge production. -High investment cost. -High energy consumption.	>99	[72,109,110,111,112,113,114,115]
Membrane Filtration	-Easy operational -technique. -High arsenate removal efficiency. -No sludge production.	-Membrane fouling. -High investment cost.	>99	[89,106,116,117,118,119,120,121,122,123,124,125,126]

**Table 4:** List of NF membranes used in arsenic removal.

NF Membrane	Materials	Arsenic Rejection (%)		References
		Arsenate	Arsenite	
**Commercial NF Membranes**				
NF-45	Porous polyamide thin-film nanocomposite (TFC) membrane	90	10–20	[149]
ES-10	Aromatic polyamide	87–93	50–89	[150]
NTR-7450	Sulfonated polyethersulfone	80.5–84.5	---	[151]
UTC-70	Polypiperazine-amide	>95	---	[151]
NF-300	TFC polyamide	60–99	---	[152]
NF-1, NF-2 and NF-20	TFC polyamide	50–100	---	[153]
NF-300	TFC polyamide	>95	---	[154]
ESNA-1-LF	Composite Polyamide	>94	---	[155]
HODRA-CORE	Sulfonated polyethersulfone	<47	---	[155]
NF-1	Aromatic polyamide	93–98	---	[156]
---	TFC membrane with aromatic polyamide Selective layer	40	---	[157]
---	Aromatic Polyamide and a polysulphone sublayer supported by nonwoven polyester structure	92–94	---	[158]
DL/DK	Polyamide layers on polyester and polysulfone support	55.8–76.2	---	[159]
NF90-4040	Polyamide TFC	94	90	[160]
Dow/FilmTec NF90	Polyamide	98	---	[161]
Dow/FilmTec NF270	Polyamide	94	---	[161]
FilmTec NF45	Aromatic Polyamide TFC	60–90	---	[162]
FilmTec NF70	TFC membrane	97	---	[121]
**Synthesized NF Membranes**				
NF-PS-3	Microporous polysulfone membrane	98.5	70.4	[163]
PMIA	Poly m–phenylene isophthalamide	>90	---	[164]
SPEEK	Sulfonated poly(ether ether ketone)	>95	---	[165]
TFN	0.15 wt% UiO-66 (MOF)	98.6	---	[166]
TFN-0.05	0.05 wt% sodium ion modified carbon quantum dot (Na-CQD) incorporated thin-film nanocomposite	99.5	---	[167]
TFC-50	50 wt% P[MPC-co-AEMA] co-polymer incorporated into polyamide selective layer of TFC membranes	99.8	---	[168]
PA-CSBF	Polyamide intercalated nanofiltration membrane modified with biofunctionalized core shell composite	---	99	[169]
AAF–NF	TFN nanofiltration membranes with aromatic amine-functionalized multiwalled carbon nanotubes	91	---	[170]
AF–NF	TFN nanofiltration membranes with aliphatic amine-functionalized multiwalled carbon nanotubes	>72	---	[170]

**Table 5:** Effect of different parameters on the rejection of arsenic by NF membranes.

NF Membrane	Arsenic Rejection (%)				References
	Arsenate [As^V^]		Arsenite [As^III^]		
Effect of arsenic concentration of feed water					
BQ01	60 at 10 µg/L	90 at 316 µg/L	28 at 10 µg/L	5 at 316 µg/L	[212]
NF-300	86 at 80 µg/L	99 at 370 µg/L	---		[213]
NF-90	Unaffected		53 at 50 µg/L	59 at 250 µg/L	[162]
NF-200	Unaffected		23 at 50 µg/L	25 at 250 µg/L	[162]
---	90 at 20 µg/L	100 at 90 µg/L	9.8 at 20 µg/L	2.0 at 90 µg/L	[214]
NE 90	90 at 20 µg/L	96 at 100 µg/L	44 at 20 µg/L	40 at 100 µg/L	[215]
NF-PS-3	88.3 at 50 ppb	97.3 at 1000 ppb	32.6 at 50 ppb	51.8 at 1000 ppb	[163]
NF-90	97 at 100 ppb	99 at 1000 ppb	---		[216]
N30F	79 at 100 ppb	74 at 1000 ppb	---		[216]
NTR-7450	80.5 at 30 µg/L	84.5 at 150 µg/L	---		[151]
UTC-70	95 at 30 µg/L	99 at 150 µg/L	---		[151]
NF90-4040	93 at 100 µg/L	90 at 1000 µg/L	90 at 100 µg/L	82 at 1000 µg/L	[160]
**Effect of pH of feed water**					
NF-45	25% at pH 4	>80% at pH 9	Unaffected		[162]
BQ01	8% at pH 4.5	85% at pH 8.5	Unaffected		[212]
NE 90	80% at pH 4	98% at pH 10	40% at pH 8	65% at pH 10	[215]
NF-PS-3	72.3% at pH 3	98.5% at pH 10	53% at pH 3	80.6% at pH 10	[163]
N30F	74% at pH 3.4	88% at pH 10	---	---	[216]
NF-90	94% at pH 3.4	98.4% at pH 10	---	---	[216]
NTR-7450	61% at pH 3	84% at pH 11	13% at pH 3	55% at pH 11	[151]
UTC-70	91% at pH 3	99.2% at pH 11	70% at pH 3	92% at pH 11	[151]
NF-1	87% at pH 3	99% at pH 10	50% at pH 3	76% at pH 10	[153]
NF-2	82% at pH 3	96% at pH 10	33% at pH 3	69% at pH 10	[153]
NF-20	86% at pH 3	98% at pH 10	43% at pH 3	71% at pH 10	[153]
Alfa Laval NF	80% at pH 6	92% at pH 8	---	---	[217]
Dow NF270	87% at pH 6	89% at pH 8	---	---	[217]
PMIA	83% at pH 3	99% at pH 9	---	---	[164]
SPEEK	89% at pH 4	96.4% at pH 9	---	---	[165]
**Effect of applied pressure**					
NF-70	97.3 at 0.4 MPa	97.5 at 0.8 MPa	96.1 at 0.4 MPa	96.3 at 0.8 MPa	[121]
ES-10	95 at 0.3 MPa	97.5 at 1.1 MPa	60 at 0.3 MPa	80 at 1.1 MPa	[218]
NTR-729HF	91 at 0.3 MPa	94 at 1.1 MPa	10 at 0.3 MPa	24 at 1.1 MPa	[218]
NTR-7250	85.5 at 0.3 MPa	86 at 1.1 MPa	8 at 0.3 MPa	15 at 1.1 MPa	[218]
NF-300	92 at 0.31 MPa	94 at 0.724 MPa	---		[213]
NF-90	Unaffected		50 at 5 bar	63 at 20 bar	[219]
NF-200	Unaffected		16 at 5 bar	30 at 20 bar	[219]
NF-PS-3	Unaffected		41 at 80 psi	56.5 at 180 psi	[163]
NF-300	90 at 10 bar	99 at 50 bar	---		[220]
NTR-7450	74 to at 0.2 MPa	82.5 at 1.0 MPa	13.5 at 0.2 MPa	21.5 at 1.0 MPa	[151]
UTC-70	95 at 0.2 MPa	99.2 at 1.0 MPa	70 at 0.2 MPa	80.5 at 1.0 MPa	[151]
NF90-4040	86 at 4 bar	94 at 7 bar	82 at 4 bar	89 at 7 bar	[160]
**Effect of temperature**					
NF-70	97.5 at 15 °C	97 at 25 °C	96 at 15 °C	96.5 at 25 °C	[121]
NF-90	99.9 at 15 °C	98.6 at 35 °C	---		[219]
NF-200	98.5 at 15 °C	96.9 at 35 °C	---		[219]
NF-90	95.4 at 15 °C	93.1 at 40 °C	---		[216]
N30F	86 at 15 °C	72 at 40 °C	---		[216]
NF90-4040	87 at 28 °C	82 at 37 °C	86 at 28 °C	81 at 37 °C	[160]
